# Discovery of significant variants containing large deletions in the 5'UTR of human hepatitis C virus (HCV)

**DOI:** 10.1186/1743-422X-3-82

**Published:** 2006-09-29

**Authors:** Dennis Revie, Michael O Alberti, Ravi S Braich, David Bayles, John G Prichard, S Zaki Salahuddin

**Affiliations:** 1 Department of Biology, California Lutheran University, Thousand Oaks, California, USA; 2 California Institute of Molecular Medicine, Ventura, California, USA; 3 Ventura County Medical Center, Ventura, California, USA; 4 Alnylam Pharmaceuticals, Cambridge, Massachusetts, USA

## Abstract

We recently reported the isolation and *in vitro *replication of hepatitis C virus. These isolates were termed CIMM-HCV and analyzed to establish genotypes and subtypes, which are reported elsewhere. During this analysis, an HCV isolated from a patient was discovered that had large deletions in the 5'UTR. 57% of the HCV RNA found in this patient's sera had 113 or 116 bp deletions. Sequence data showed that domains IIIa to IIIc were missing. Previous studies have suggested that these domains may be important for translation. *In vitro *replicated HCV from this patient did not contain these deletions, however, it contained a 148 bp deletion in the 5'UTR. Whereas the patient HCV lacked domains IIIa through IIIc, the isolate lacked domains IIIa through IIId. HCV from this patient continues to produce large deletions *in vitro*, suggesting that the deletion may not be important for the assembly or replication of the virus. This is the first report describing these large deletions.

## Background

HCV is a cause of several serious diseases, and is estimated to infect around 3% of the world's population [1]. This virus contains a 5'UTR, which is a conserved 341 nucleotide stretch. This region has been used to establish the major HCV genotypes [2,3]. Other regions of the HCV genome have been used to help determine subtypes. Among the major genotypes, up to 30% of the sequences of the major HCV strains can differ from each other [4].

Synthetic sequences called Replicons have been used to study the functional aspects of the 5'UTR. This contains the IRES region, which is important for the translation of HCV RNA. Three domains: I, II, and III, are inside this region. Domains I and II were shown to be important for Replicon multiplication [5,6], and deletions of parts of domain III can reduce the *in vitro *translation efficiency [7]. Spahn *et al*. [8] used cryoelectron microscopy to show that the 40S ribosomal subunit binds to domain III, and the translation initiation factor eIF-3 binds to domain IIIb [9]. A number of other proteins have been reported to bind to the IRES, as well. This information has been obtained using Replicons, which were developed by Bartenschlager and his associates.

We have developed an *in vitro *system that can isolate and replicate HCV [10]. While studying HCV isolated by this system, we discovered a patient that was missing part of the 5'UTR. This report describes these large deletions.

## Results

Patient 313 is a 51 year old woman with long-standing HCV chronic-active hepatitis that developed porphyria cutanea tarda which required intermittent phlebotomy for symptomatic relief and prevention. Otherwise, she was in good health and had not undergone liver biopsy procedure or any HCV treatment before the blood sample was obtained. There was no history of hepatitis B virus or HIV-1 infection. Phlebotomy was performed by standard techniques using transfusion donor bags containing sodium heparin. We received this anti-coagulated sample for use in HCV investigations.

**Figure 1:**
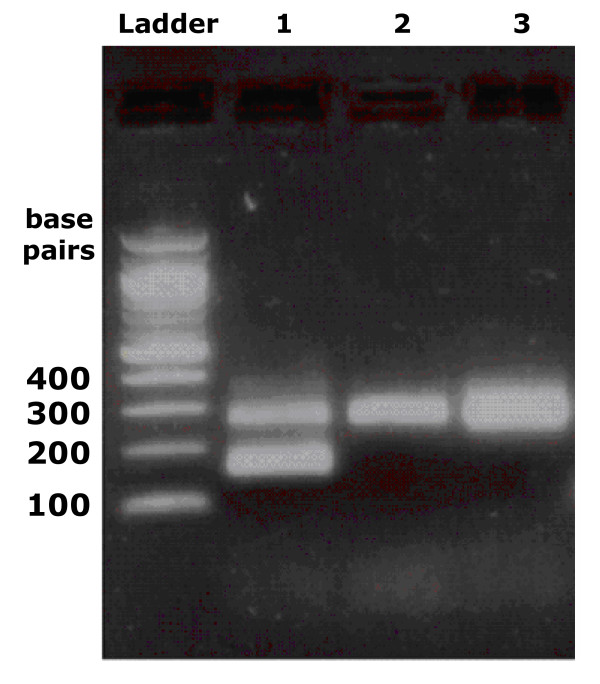
**Analysis of deletions found in patient 313 sera and CIMM-HCV isolates. Agarose gel electrophoresis of RT-PCR products of HCV RNA isolated from plasma and *in vitro *cultures**. A 100 bp ladder was used (NE Biolabs). Lane 1 is 313 plasma, lane 2 is 313-i, lane 3 is 313-T1. The 269 bp fragment contains the 5'UTR. The approximately 150 bp fragments observed in 313 plasma were shown by sequencing to contain large deletions of the 5'UTR.

Gel electrophoresis of RT-PCR fragments of the 5'UTR for patient 313 showed two bands on agarose gels instead of the single band that is normally observed (Figure 1, Lane 1). To determine why there were two bands, we cloned and sequenced the PCR fragments (Table 1).

**Table 1:** List of CIMM-HCV isolates cloned and sequenced

**Isolate**	**Number of clones sequenced**	**Genbank accession numbers**	**Description**
313 plasma	60	EF028185	Patient sample
313-i	26	EF028184	Primary isolate in macrophages
313-T1	26	EF028187	Secondary isolate in B-cells
313-T1b	32	EF028188	Secondary isolate, cultured 4 months longer

**Figure 2:**
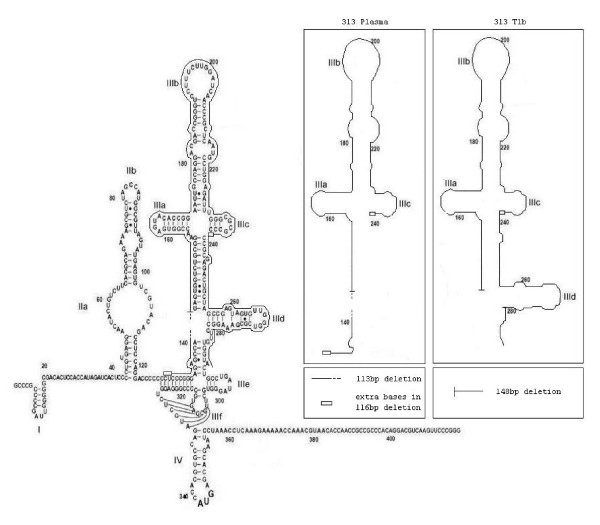
**Map of deletions found in 313 plasma and 313T1**. A 2D map of the 313 plasma consensus sequence is shown. The extents of the large deletions found in 313 plasma are shown by the outlining. The inset shows the extent of the large deletions found in 313-T1b. This figure is adapted from Lyons *et al*. [23]. The 5'UTR sequences of the samples containing deletions have the accession numbers EF028186, EF028194, and EF028189.

Analysis of HCV sequences from patient 313 showed that 57% of the clones contained either a 113 or a 116 bp deletion (Figure 1, Lane 1). The deleted regions extended from either bases 126 or 129 to 241. The deletions of 113 or 116 bp were limited to the region between two strings of C's, with 56% of the sequences containing deletions lacking 113 bp and 44% lacking 116 bp. Most of the IRES, including loops IIIa, IIIb, and IIIc, was missing (Figure 2). The consensus sequence of the 313 plasma HCV that contained the deletions was the same as the consensus sequence for the full length 313 plasma HCV, except for the deletion.

The HCV was isolated from the patient in the usual manner as was reported before [11]. The HCV produced by macrophages is called the primary isolate and is designated 313-i. A cell-free supernatant of this isolate is then used to infect Epstein-Barr virus transformed B-cells. The HCV produced by B-cells is the secondary isolate, and designated 313-T1.

We found no deletions in the primary isolate 313-i (Figure 1, Lane 2). There were no deletions in the first 313-T1 sample analyzed (Figure 1, Lane 3), but 6 clones had deletions in 313-T1b (19%). 313-T1 was isolated on 10/18/04 and 313-T1b was isolated on 2/24/05, so 313-T1b was grown in culture four months longer than 313-T1. This deletion extends from bases 141 to 288, and is therefore a different deletion than the one found in the patient's blood (Figure 2).

We also noted that each of the 313 samples had at least one clone that contained an extra C in the string of C's from bases 120 to 126. Other isolates from different patients sometimes also contain an extra C in this region [11].

## Discussion

We have previously reported the isolation of HCV from infected patients and *in vitro *replication of these isolates [10]. A molecular analysis of CIMM-HCV for possible subtypes and quasispecies was recently performed which showed that the isolated HCV had only minor sequence changes compared to patient HCV [11].

A patient with unique deletions is the subject of this study. This patient had not yet undergone therapy, and therefore the deletions found in the patient were not induced by treatment. Deletions of up to 18 bases in the 5'UTR, along with additions of up to 40 bases have previously been reported [12], and deletions of up to 2 kb have been found in the protein coding region of HCV [13]. The deletions of 113 or 116 bp in patient 313 were limited to the region between two strings of C's in the 5'UTR. Domains IIIa through IIIc, which are missing in these deletions, are thought to be bound by the right leg of eIF3 [14]. Otto *et al*. [15] crosslinked a IIIa to IIIc domain deletion named del_IIIabc to the 40S ribosomal subunit. Del_IIIabc, which lacks bases 152 to 240, crosslinked to the small ribosomal subunit proteins to about the same extent as did the wild-type IRES. This suggests that the deletion found in patient 313 would probably bind to the 40S subunit. An alternative mechanism of initiation of translation in high MgCl_2 _was recently reported for HCV [16]. This pathway does not require eIF3 or other known translation initiation factors. It may be that the deletions we are reporting cause this alternate method of initiation to be used. Although deletions in domain III may adversely affect the translation of HCV RNA, they do not appear to affect HCV replication. This may be because only domains I and II are needed for this purpose [6]. It is possible that the deletions of stem loops in domain III may reduce *in vitro *translation [6,7,17-22]. It is also possible that wild type 313 HCV without the deletion is infectious, but in the process of replication produces a large amount of defective, non-infectious virus particles.

Although the 113 or 116 bp deletions were found in patient 313's blood, the primary isolate, 313-i, did not contain these deletions. This may be due to selection by the macrophages. The deletion that we found in 313-T1b appears to have occurred during the culturing, as the size of 148 bp and its location in the 5'UTR are not the same as those found in the patient's blood. Since the 148 bp region lacks domains IIIa to IIId, it is unlikely to be efficiently used for translation. Only the 313 isolate and not isolates from other patients produced the deleted version *in vitro*. A mutation in the polymerase gene could decrease fidelity or processivity. Sequencing the entire genome of both the 313-T1 and the HCV found in the patient's blood may be helpful in understanding this phenomenon. Papers that have reported HCV sequences often analyze the hyper-variable region, therefore, they would have not detected these deletions. Other researchers have used one or both PCR primers that are either inside or overlap the region of the deletion, so they also would have not been detected. For this study, we cloned the entire PCR reactions to ensure that all possible HCV sequences were detected.

The presence of an extra C in the 5'UTR region was found in the HCV RNA from the 313 plasma sample and for 313T1 and three isolates from patient 081 [11]. Plasma from patient 313 contains the extra C, which could cause the HCV RNA polymerase to accidentally create the deleted versions of the 5'UTR. However, of our isolates containing the extra C's, only 313T1 had variants with large deletions, but that particular deletion was not located adjacent to that extra C. Others have reported the effects on translation of particular regions of the 5' and 3' ends of the 5'UTR containing deletions [17,18]. These and other reports, however, do not cover the same regions as we have noted here.

These isolates of HCV containing large deletions should prove useful in understanding translation of HCV RNA.

## Methods

The *in vitro *culture system, RT-PCR, sequencing, and bioinformatics were performed as described in Revie *et al*. [11]. The 313 isolates have the GenBank accession numbers of EF028185, EF028184, EF028187, and EF028188.

## Declaration of competing interests

All intellectual rights are reserved by the California Institute of Molecular Medicine (CIMM), and all aspects of this work were performed by CIMM. There are no competing interests between California Lutheran University or any other body and CIMM.

## Authors' contributions

SZS performed the biological work and the isolations, transmissions, and retransmissions of HCV. JGP performed the clinical work, recruitment of patients, and procurement of specimens. DR, MOA, RSB, and DB performed the molecular work.
